# 1-Methyl-4-[(*E*)-2-(2-thien­yl)ethen­yl]pyridinium 4-chloro­benzene­sulfonate^1^

**DOI:** 10.1107/S1600536808003929

**Published:** 2008-02-13

**Authors:** Suchada Chantrapromma, Chotika Laksana, Pumsak Ruanwas, Hoong-Kun Fun

**Affiliations:** a Department of Chemistry, Faculty of Science, Prince of Songkla University, Hat-Yai, Songkhla 90112, Thailand; b X-ray Crystallography Unit, School of Physics, Universiti Sains Malaysia, 11800 USM, Penang, Malaysia

## Abstract

In the title compound, C_12_H_12_NS^+^·C_6_H_4_ClO_3_S^−^, the cation is almost planar and exists in the *E* configuration. The cations and anions form alternate layers parallel to the *ab* plane. Within each layer, both cations and anions form chains directed along the *b* axis. The mol­ecules are inter­connected by weak C—H⋯O inter­actions into a three-dimensional network. The crystal structure is further stabilized by C—H⋯π inter­actions involving the thio­phene ring. The sulfonate and thio­phene groups are involved in weak intra­molecular C—H⋯O and C—H⋯S inter­actions, respectively. The latter intra­molecular hydrogen bonds produce *S*(5) ring motifs.

## Related literature

For bond lengths and angles, see Allen (2002 ▶); Allen *et al.* (1987 ▶). For related literature on hydrogen-bond motifs, see Bernstein *et al.* (1995 ▶). For related structures, see for example Chantrapromma *et al.* (2005 ▶, 2006*a* ▶,*b* ▶, 2007*a* ▶,*b* ▶,*c* ▶,*d* ▶); Drost *et al.* (1995 ▶); Jindawong *et al.* (2005 ▶); Subramaniyan *et al.* (2003 ▶).
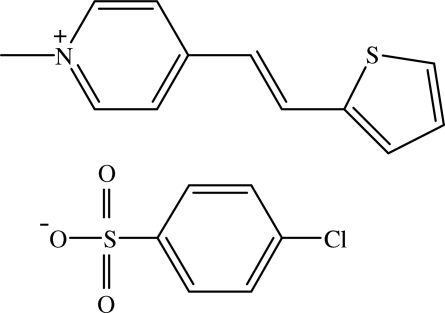


## Experimental

### 

#### Crystal data

C_12_H_12_NS^+^·C_6_H_4_ClO_3_S^−^
*M* *_r_* = 393.91
Monoclinic, 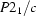

*a* = 7.3532 (1) Å
*b* = 14.0250 (2) Å
*c* = 18.3755 (2) Å
β = 111.232 (1)°
*V* = 1766.41 (4) Å^3^
*Z* = 4
Mo *K*α radiation
μ = 0.47 mm^−1^
*T* = 100.0 (1) K
0.49 × 0.22 × 0.18 mm

#### Data collection

Bruker SMART APEX2 CCD area-detector diffractometer
Absorption correction: multi-scan (*SADABS*; Bruker, 2005 ▶) *T*_min_ = 0.883, *T*_max_ = 0.919
23692 measured reflections
4688 independent reflections
3913 reflections with *I* > 2σ(*I*)
*R*_int_ = 0.037

#### Refinement

*R*[*F* ^2^ > 2σ(*F* ^2^)] = 0.037
*wR*(*F* ^2^) = 0.094
*S* = 1.06
4688 reflections
227 parameters
H-atom parameters constrained
Δρ_max_ = 0.57 e Å^−3^
Δρ_min_ = −0.42 e Å^−3^

### 

Data collection: *APEX2* (Bruker, 2005 ▶); cell refinement: *APEX2*; data reduction: *SAINT* (Bruker, 2005 ▶); program(s) used to solve structure: *SHELXTL* (Sheldrick, 2008 ▶); program(s) used to refine structure: *SHELXTL*; molecular graphics: *SHELXTL*; software used to prepare material for publication: *SHELXTL* and *PLATON* (Spek, 2003 ▶).

## Supplementary Material

Crystal structure: contains datablocks global, I. DOI: 10.1107/S1600536808003929/fb2086sup1.cif

Structure factors: contains datablocks I. DOI: 10.1107/S1600536808003929/fb2086Isup2.hkl

Additional supplementary materials:  crystallographic information; 3D view; checkCIF report

## Figures and Tables

**Table 1:** Hydrogen-bond geometry (Å, °) *Cg1* is the centroid of the S1/C8–C11 thiophene ring.

*D*—H⋯*A*	*D*—H	H⋯*A*	*D*⋯*A*	*D*—H⋯*A*
C3—H3*A*⋯O3^i^	0.93	2.31	3.211 (2)	164
C6—H6*A*⋯S1	0.93	2.84	3.228 (2)	106
C7—H7*A*⋯O1^ii^	0.93	2.39	3.266 (2)	157
C9—H9*A*⋯O3^ii^	0.93	2.58	3.495 (2)	166
C10—H10*A*⋯O2^iii^	0.93	2.39	3.302 (2)	167
C11—H11*A*⋯O2^iv^	0.93	2.55	3.063 (2)	115
C12—H12*C*⋯O2^i^	0.96	2.39	3.334 (2)	168
C17—H17*A*⋯O2^iv^	0.93	2.41	3.318 (2)	166
C18—H18*A*⋯O3	0.93	2.51	2.892 (2)	105
C12—H12*B*⋯*Cg*1^i^	0.96	2.69	3.515 (2)	144

Symmetry codes: (i) 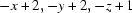; (ii) 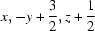; (iii) 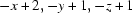; (iv) 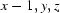.

**Fig. 1.**
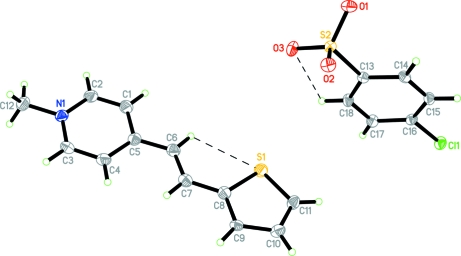
The title molecules showing 50% probability displacement ellipsoids and the atom-numbering scheme. The intramolecular C—H···O and C—H···S weak interactions are drawn as dashed lines.

**Fig. 2.**
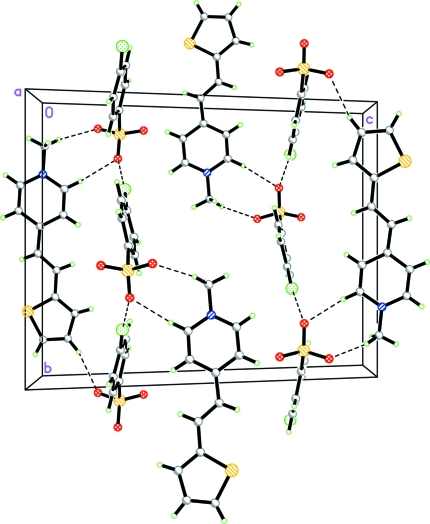
The packing diagram of the title structure viewed along the a axis. The weak C—H···O and C—H···S interactions are drawn as dashed lines.

## supplementary crystallographic information

### Comment

Molecules with extended π systems have been extensively used in attempts to obtain materials with non-linear optical (NLO) properties. Organic crystal with the required conjugated π electrons are attractive candidates because of the large NLO coefficients. In our research for second-order NLO materials, we have previously synthesized and crystallized several organic ionic salts of pyridinium and quinolinium derivatives to study their non-linear optical properties (Chantrapromma *et al.*, 2005; 2006*a*; 2006*b*; 2007*a*; 2007*b*; 2007*c*; 2007 d; Jindawong *et al.*, 2005). An earlier study carried out by Drost *et al.* (1995) has revealed that the products of the dipole moment and the molecular hyperpolarizability (β) of thiophene-containing conjugated moieties are larger than those of the phenyl analogues. Based on this reason, we have synthesized the title compound which was designed by replacement of the cationic phenyl ring by the thiophene ring that is present in 4-(4'-Hydroxy-3'-methoxystyryl)-1-methylpyridinium 4-chlorobenzenesulfonate (Chantrapromma *et al.*, 2005).

The asymmetric unit of the title compound consists of the C_12_H_12_NS^+^ cation and the C_6_H_4_ClO_3_S^-^ anion. The cation is almost planar and exists in the *E* configuration with respect to the C6?C7 double bond [1.334 (3) Å]. The cation is almost perpendicular to the anion as is indicated by the angles between the mean planes of the chlorophenyl ring to the pyridinium as well as to the thiophene ring being 87.64 (9)° and 86.73 (9)°, respectively. The dihedral angle between the pyridinium and the thiophene rings is 5.74 (10)°. The ethenyl unit is nearly planar. The torsion angles C4–C5–C6–C7 = -4.3 (3)° and C6–C7–C8–S1 = -1.5 (3)°.

The atom O3 of the sulfonate and the S atom of the thiophene contribute to the C—H···O and C—H···S intramolecular weak interactions (Fig. 1 and Table 1) forming S(5) ring motifs (Bernstein *et al.*, 1995). The bond lengths and angles are normal (Allen *et al.*, 1987) and are comparable with closely related structures (Chantrapromma *et al.*, 2005; 2006*b*; 2007*c*; 2007 d).

All the O atoms of 4-chlorobenzenesulfonate anion are involved in the C—H···O weak interactions (Table 1). The cations and anions form alternate layers parallel to the *ab* plane. Within each respective layer, the ions are interconnected by C—H···O weak interactions and in each respective layer can be distinguished chains directed along the *b* axis. The alternating layers are separated by 4.282 (2) Å and are further linked into a three dimensional network by C—H···O weak interactions (Table 1). The sulfonate as well as the thiophene are involved in C—H···O and C—H···S intramolecular weak interactions, respectively. These weak hydrogen bonds participate in S(5) ring motifs. The crystal structure is further stabilized by the C12—H12B···π interaction to the thiophene ring C8–C11/S1: C12—H12B=0.96; H12B···*Cg*1^i^=2.692; C12—*Cg*1^i^=3.515 (2) Å; C12—H12B···*Cg*1^i^= 144°. [*Cg*1^i^ is the centroid of the S1/C8–C11 thiophene ring (symmetry code: (i): 2 - *x*, 2 - *y*, 1 - *z*).]

A very interesting feature is the short non-bonding contact between Cl1 and O3 that is 2.963 (1) Å long only. A search in the Cambridge Structural Database (version 5.29 and addenda up to 25-th January 2008; Allen, 2002) among the structures which have been flagged with no error or disorder as well as with *R*-factor < 0.05 and which contained chloro-phenyl with any substituent in the *para* position showed that the present structure contains an unprecedentedly short contact of this kind. The up-to-now shortest contact of this type was 2.996 (2) Å long and it was observed in MUTDOU [Spiro(2-carbomethoxy-3-(4-chlorophenyl)-5-(*S*,*R*)-(*cis*-1-(4-methoxyphenyl) -3-phenyl-4-oxoazetidin-2-(S,*R*)-yl)pyrrolidine-4,3^1^-chroman-4^1^-one)] determined by Subramaniyan *et al.* (2003).

### Experimental

4-(2-Thiophenestyryl)-1-methylpyridinium iodide (compound A) was synthesized by mixing a solution (1:1:1 molar ratio) of 1,4-dimethylpyridinium iodide (2.00 g, 8.5 mmol), 2-thiophenecarboxaldehyde (6.00 ml, 8.5 mmol) and piperidine (0.84 ml, 8.5 mmol) in hot methanol (40 ml). The resulting solution was refluxed for 5 h under a nitrogen atmosphere. The resultant solid was filtered off, washed with diethyl ether and recrystallized from methanol. The title compound was synthesized by mixing compound A (0.10 g, 0.3 mmol) in hot methanol (20 ml) and silver(I) 4-chlorobenzenesulfonate (0.08 g, 0.3 mmol) in hot methanol (30 ml). Silver(I) 4-chlorobenzenesulfonate was synthesized according to our previously reported procedure (Chantrapromma *et al.*, 2006*b*). The mixture immediately yielded a grey solid of silver iodide. After stirring the mixture for *ca* 30 min, the precipitate of silver iodide was removed and the resulting solution was evaporated and a brown solid was obtained. Brown block-shaped single crystals of the title compound suitable for *x*-ray structure determination were recrystalized from methanol solvent by slow evaporation of the solvent at room temperature after several days (Mp. 503–505 K).

### Refinement

All the hydrogen atoms could have been discerned in the difference Fourier map. Nevertheless, all the H atoms attached to the carbon atoms were constrained in a riding motion approximation with C_aryl_—H=0.93 and C_methyl_—H=0.968 Å. The *U*_iso_ values were constrained to be 1.5*U*_eq_ of the carrier atom for methyl H atoms and 1.2*U*_eq_ for the remaining H atoms. A rotating group model was used for the methyl groups. The highest residual electron density peak is located at 0.75 Å from C8 and the deepest hole is located at 0.50 Å from S1.

### Crystal data

**Table:**

C_12_H_12_NS^+^·C_6_H_4_ClO_3_S^–^	*F*_000_ = 816
*M**_r_* = 393.91	*D*_x_ = 1.481 Mg m^−^^3^
Monoclinic, *P*2_1_/*c*	Melting point = 503–505 K
Hall symbol: -P 2ybc	Mo *K*α radiation λ = 0.71073 Å
*a* = 7.3532 (1) Å	Cell parameters from 4688 reflections
*b* = 14.0250 (2) Å	θ = 1.9–29.0º
*c* = 18.3755 (2) Å	µ = 0.47 mm^−^^1^
β = 111.232 (1)º	*T* = 100.0 (1) K
*V* = 1766.41 (4) Å^3^	Block, brown
*Z* = 4	0.49 × 0.22 × 0.18 mm

### Data collection

**Table:**

Bruker SMART APEX2 CCD area-detector diffractometer	4688 independent reflections
Radiation source: fine-focus sealed tube	3913 reflections with *I* > 2σ(*I*)
Monochromator: graphite	*R*_int_ = 0.037
Detector resolution: 8.33 pixels mm^-1^	θ_max_ = 29.0º
*T* = 100.0(1) K	θ_min_ = 1.9º
ω scans	*h* = −10→10
Absorption correction: multi-scan(SADABS; Bruker, 2005)	*k* = −19→19
*T*_min_ = 0.883, *T*_max_ = 0.919	*l* = −21→25
23692 measured reflections	

### Refinement

**Table:**

Refinement on *F*^2^	Secondary atom site location: difference Fourier map
Least-squares matrix: full	Hydrogen site location: difference Fourier map
*R*[*F*^2^ > 2σ(*F*^2^)] = 0.037	H-atom parameters constrained
*wR*(*F*^2^) = 0.094	*w* = 1/[σ^2^(*F*_o_^2^) + (0.0415*P*)^2^ + 1.1208*P*] where *P* = (*F*_o_^2^ + 2*F*_c_^2^)/3
*S* = 1.06	(Δ/σ)_max_ = 0.001
4688 reflections	Δρ_max_ = 0.57 e Å^−^^3^
227 parameters	Δρ_min_ = −0.42 e Å^−^^3^
63 constraints	Extinction correction: none
Primary atom site location: difference Fourier map	

### Special details

**Table:**

Experimental. The low-temparture data was collected with the Oxford Cryosystem Cobra low-temperature attachment.
Geometry. All e.s.d.'s (except the e.s.d. in the dihedral angle between two l.s. planes) are estimated using the full covariance matrix. The cell e.s.d.'s are taken into account individually in the estimation of e.s.d.'s in distances, angles and torsion angles; correlations between e.s.d.'s in cell parameters are only used when they are defined by crystal symmetry. An approximate (isotropic) treatment of cell e.s.d.'s is used for estimating e.s.d.'s involving l.s. planes.
Refinement. Refinement of *F*^2^ against ALL reflections. The weighted *R*-factor *wR* and goodness of fit *S* are based on *F*^2^, conventional *R*-factors *R* are based on *F*, with *F* set to zero for negative *F*^2^. The threshold expression of *F*^2^ > σ(*F*^2^) is used only for calculating *R*-factors(gt) *etc*. and is not relevant to the choice of reflections for refinement. *R*-factors based on *F*^2^ are statistically about twice as large as those based on *F*, and *R*- factors based on ALL data will be even larger.

### Fractional atomic coordinates and isotropic or equivalent isotropic displacement parameters (Å^2^)

**Table:**

	*x*	*y*	*z*	*U*_iso_*/*U*_eq_	
S1	0.57131 (7)	0.72654 (3)	0.43805 (3)	0.02450 (11)	
S2	1.05267 (5)	0.60506 (3)	0.27447 (2)	0.01477 (10)	
Cl1	0.37650 (6)	0.31936 (3)	0.25045 (2)	0.02092 (10)	
O1	1.09007 (17)	0.58763 (9)	0.20315 (7)	0.0219 (3)	
O2	1.21870 (16)	0.58102 (9)	0.34443 (7)	0.0196 (3)	
O3	0.97435 (17)	0.69943 (8)	0.27824 (7)	0.0210 (3)	
N1	0.8323 (2)	1.24164 (11)	0.49389 (9)	0.0214 (3)	
C1	0.6981 (3)	1.10669 (14)	0.41625 (11)	0.0273 (4)	
H1A	0.6342	1.0808	0.3669	0.033*	
C2	0.7408 (3)	1.20156 (14)	0.42351 (11)	0.0265 (4)	
H2A	0.7060	1.2394	0.3790	0.032*	
C3	0.8863 (3)	1.18711 (14)	0.55895 (11)	0.0259 (4)	
H3A	0.9501	1.2150	0.6075	0.031*	
C4	0.8481 (3)	1.09132 (14)	0.55424 (11)	0.0267 (4)	
H4A	0.8881	1.0547	0.5995	0.032*	
C5	0.7490 (2)	1.04783 (13)	0.48184 (11)	0.0216 (3)	
C6	0.6999 (3)	0.94698 (14)	0.47236 (11)	0.0239 (4)	
H6A	0.6262	0.9252	0.4226	0.029*	
C7	0.7537 (3)	0.88364 (13)	0.53044 (11)	0.0227 (4)	
H7A	0.8268	0.9066	0.5799	0.027*	
C8	0.7098 (2)	0.78300 (13)	0.52426 (10)	0.0213 (3)	
C9	0.7700 (2)	0.72058 (12)	0.58541 (10)	0.0190 (3)	
H9A	0.8451	0.7379	0.6363	0.023*	
C10	0.7037 (3)	0.62467 (13)	0.56186 (11)	0.0234 (4)	
H10A	0.7313	0.5730	0.5958	0.028*	
C11	0.5956 (3)	0.61854 (13)	0.48386 (11)	0.0234 (4)	
H11A	0.5415	0.5622	0.4586	0.028*	
C12	0.8779 (3)	1.34470 (13)	0.50102 (12)	0.0287 (4)	
H12A	0.8119	1.3754	0.4518	0.043*	
H12B	1.0161	1.3535	0.5159	0.043*	
H12C	0.8353	1.3722	0.5400	0.043*	
C13	0.8648 (2)	0.52345 (12)	0.27171 (9)	0.0151 (3)	
C14	0.8824 (2)	0.42781 (12)	0.25482 (9)	0.0165 (3)	
H14A	0.9944	0.4067	0.2475	0.020*	
C15	0.7332 (2)	0.36386 (12)	0.24892 (10)	0.0174 (3)	
H15A	0.7434	0.3000	0.2372	0.021*	
C16	0.5682 (2)	0.39778 (12)	0.26093 (9)	0.0165 (3)	
C17	0.5512 (2)	0.49151 (12)	0.28055 (10)	0.0186 (3)	
H17A	0.4417	0.5120	0.2902	0.022*	
C18	0.7013 (2)	0.55480 (12)	0.28563 (10)	0.0181 (3)	
H18A	0.6920	0.6183	0.2984	0.022*	

### Atomic displacement parameters (Å^2^)

**Table:**

	*U*^11^	*U*^22^	*U*^33^	*U*^12^	*U*^13^	*U*^23^
S1	0.0255 (2)	0.0248 (2)	0.0196 (2)	−0.00284 (17)	0.00382 (17)	0.00120 (17)
S2	0.01233 (18)	0.01553 (19)	0.01604 (19)	0.00021 (14)	0.00465 (14)	0.00095 (14)
Cl1	0.01861 (19)	0.0189 (2)	0.0272 (2)	−0.00466 (14)	0.01069 (16)	−0.00201 (16)
O1	0.0200 (6)	0.0292 (7)	0.0181 (6)	−0.0020 (5)	0.0090 (5)	0.0007 (5)
O2	0.0144 (5)	0.0239 (6)	0.0174 (6)	0.0000 (4)	0.0022 (5)	0.0013 (5)
O3	0.0181 (6)	0.0143 (6)	0.0314 (7)	−0.0003 (4)	0.0099 (5)	0.0002 (5)
N1	0.0225 (7)	0.0214 (7)	0.0240 (8)	−0.0001 (6)	0.0127 (6)	0.0011 (6)
C1	0.0289 (9)	0.0304 (10)	0.0204 (9)	−0.0058 (8)	0.0063 (7)	−0.0019 (7)
C2	0.0298 (10)	0.0285 (10)	0.0192 (8)	−0.0031 (8)	0.0066 (7)	0.0034 (7)
C3	0.0309 (9)	0.0293 (10)	0.0174 (8)	−0.0016 (8)	0.0085 (7)	−0.0019 (7)
C4	0.0329 (10)	0.0273 (10)	0.0208 (9)	0.0009 (8)	0.0108 (8)	0.0035 (7)
C5	0.0192 (8)	0.0227 (9)	0.0256 (9)	−0.0001 (6)	0.0114 (7)	−0.0001 (7)
C6	0.0219 (8)	0.0285 (9)	0.0209 (8)	−0.0034 (7)	0.0073 (7)	−0.0016 (7)
C7	0.0192 (8)	0.0274 (9)	0.0224 (9)	0.0001 (7)	0.0086 (7)	−0.0019 (7)
C8	0.0181 (8)	0.0239 (9)	0.0227 (8)	−0.0015 (6)	0.0085 (7)	−0.0015 (7)
C9	0.0176 (8)	0.0251 (9)	0.0133 (7)	−0.0012 (6)	0.0045 (6)	0.0008 (6)
C10	0.0219 (8)	0.0249 (9)	0.0220 (9)	−0.0019 (7)	0.0061 (7)	0.0044 (7)
C11	0.0225 (8)	0.0211 (9)	0.0242 (9)	−0.0038 (7)	0.0058 (7)	0.0004 (7)
C12	0.0318 (10)	0.0219 (9)	0.0376 (11)	−0.0020 (8)	0.0186 (9)	−0.0005 (8)
C13	0.0139 (7)	0.0171 (8)	0.0142 (7)	−0.0002 (6)	0.0048 (6)	−0.0002 (6)
C14	0.0145 (7)	0.0190 (8)	0.0170 (8)	0.0024 (6)	0.0068 (6)	−0.0008 (6)
C15	0.0186 (7)	0.0148 (7)	0.0192 (8)	0.0016 (6)	0.0075 (6)	−0.0010 (6)
C16	0.0148 (7)	0.0176 (8)	0.0166 (7)	−0.0027 (6)	0.0051 (6)	0.0008 (6)
C17	0.0148 (7)	0.0180 (8)	0.0249 (9)	0.0018 (6)	0.0094 (7)	0.0001 (6)
C18	0.0182 (8)	0.0148 (8)	0.0217 (8)	0.0011 (6)	0.0078 (6)	−0.0014 (6)

### Geometric parameters (Å, °)

**Table:**

S1—C11	1.7105 (19)	C7—C8	1.443 (3)
S1—C8	1.7335 (18)	C7—H7A	0.9300
S2—O1	1.4541 (12)	C8—C9	1.366 (2)
S2—O3	1.4550 (12)	C9—C10	1.443 (2)
S2—O2	1.4560 (12)	C9—H9A	0.9300
S2—C13	1.7806 (16)	C10—C11	1.367 (3)
Cl1—C16	1.7428 (16)	C10—H10A	0.9300
N1—C2	1.346 (2)	C11—H11A	0.9300
N1—C3	1.352 (2)	C12—H12A	0.9600
N1—C12	1.479 (2)	C12—H12B	0.9600
C1—C2	1.363 (3)	C12—H12C	0.9600
C1—C5	1.395 (3)	C13—C18	1.388 (2)
C1—H1A	0.9300	C13—C14	1.393 (2)
C2—H2A	0.9300	C14—C15	1.390 (2)
C3—C4	1.369 (3)	C14—H14A	0.9300
C3—H3A	0.9300	C15—C16	1.393 (2)
C4—C5	1.404 (3)	C15—H15A	0.9300
C4—H4A	0.9300	C16—C17	1.381 (2)
C5—C6	1.455 (3)	C17—C18	1.393 (2)
C6—C7	1.334 (3)	C17—H17A	0.9300
C6—H6A	0.9300	C18—H18A	0.9300
			
C11—S1—C8	91.87 (9)	C8—C9—C10	112.16 (15)
O1—S2—O3	113.77 (7)	C8—C9—H9A	123.9
O1—S2—O2	112.64 (7)	C10—C9—H9A	123.9
O3—S2—O2	112.86 (7)	C11—C10—C9	112.22 (16)
O1—S2—C13	105.32 (7)	C11—C10—H10A	123.9
O3—S2—C13	105.65 (7)	C9—C10—H10A	123.9
O2—S2—C13	105.68 (7)	C10—C11—S1	112.19 (14)
C2—N1—C3	119.87 (16)	C10—C11—H11A	123.9
C2—N1—C12	120.78 (16)	S1—C11—H11A	123.9
C3—N1—C12	119.34 (16)	N1—C12—H12A	109.5
C2—C1—C5	120.86 (18)	N1—C12—H12B	109.5
C2—C1—H1A	119.6	H12A—C12—H12B	109.5
C5—C1—H1A	119.6	N1—C12—H12C	109.5
N1—C2—C1	121.28 (17)	H12A—C12—H12C	109.5
N1—C2—H2A	119.4	H12B—C12—H12C	109.5
C1—C2—H2A	119.4	C18—C13—C14	120.17 (15)
N1—C3—C4	120.76 (17)	C18—C13—S2	120.39 (13)
N1—C3—H3A	119.6	C14—C13—S2	119.44 (12)
C4—C3—H3A	119.6	C15—C14—C13	120.28 (15)
C3—C4—C5	120.70 (17)	C15—C14—H14A	119.9
C3—C4—H4A	119.6	C13—C14—H14A	119.9
C5—C4—H4A	119.6	C14—C15—C16	118.36 (15)
C1—C5—C4	116.51 (17)	C14—C15—H15A	120.8
C1—C5—C6	119.63 (17)	C16—C15—H15A	120.8
C4—C5—C6	123.86 (17)	C17—C16—C15	122.20 (15)
C7—C6—C5	124.26 (17)	C17—C16—Cl1	118.91 (12)
C7—C6—H6A	117.9	C15—C16—Cl1	118.89 (13)
C5—C6—H6A	117.9	C16—C17—C18	118.65 (15)
C6—C7—C8	126.54 (17)	C16—C17—H17A	120.7
C6—C7—H7A	116.7	C18—C17—H17A	120.7
C8—C7—H7A	116.7	C13—C18—C17	120.27 (15)
C9—C8—C7	124.44 (16)	C13—C18—H18A	119.9
C9—C8—S1	111.55 (13)	C17—C18—H18A	119.9
C7—C8—S1	124.01 (14)		
			
C3—N1—C2—C1	1.0 (3)	C8—C9—C10—C11	−0.1 (2)
C12—N1—C2—C1	179.98 (18)	C9—C10—C11—S1	−0.2 (2)
C5—C1—C2—N1	−0.3 (3)	C8—S1—C11—C10	0.36 (15)
C2—N1—C3—C4	−0.4 (3)	O1—S2—C13—C18	−130.91 (14)
C12—N1—C3—C4	−179.30 (17)	O3—S2—C13—C18	−10.19 (16)
N1—C3—C4—C5	−1.1 (3)	O2—S2—C13—C18	109.65 (14)
C2—C1—C5—C4	−1.0 (3)	O1—S2—C13—C14	48.59 (15)
C2—C1—C5—C6	179.41 (18)	O3—S2—C13—C14	169.30 (13)
C3—C4—C5—C1	1.7 (3)	O2—S2—C13—C14	−70.85 (14)
C3—C4—C5—C6	−178.76 (17)	C18—C13—C14—C15	2.4 (2)
C1—C5—C6—C7	175.16 (18)	S2—C13—C14—C15	−177.09 (12)
C4—C5—C6—C7	−4.4 (3)	C13—C14—C15—C16	−0.5 (2)
C5—C6—C7—C8	−179.72 (17)	C14—C15—C16—C17	−1.9 (3)
C6—C7—C8—C9	179.23 (18)	C14—C15—C16—Cl1	177.84 (12)
C6—C7—C8—S1	−1.5 (3)	C15—C16—C17—C18	2.4 (3)
C11—S1—C8—C9	−0.44 (14)	Cl1—C16—C17—C18	−177.33 (13)
C11—S1—C8—C7	−179.82 (15)	C14—C13—C18—C17	−1.9 (2)
C7—C8—C9—C10	179.78 (16)	S2—C13—C18—C17	177.59 (13)
S1—C8—C9—C10	0.40 (19)	C16—C17—C18—C13	−0.5 (2)

### Hydrogen-bond geometry (Å, °)

**Table:**

*D*—H···*A*	*D*—H	H···*A*	*D*···*A*	*D*—H···*A*
C3—H3A···O3^i^	0.93	2.31	3.211 (2)	164
C6—H6A···S1	0.93	2.84	3.228 (2)	106
C7—H7A···O1^ii^	0.93	2.39	3.266 (2)	157
C9—H9A···O3^ii^	0.93	2.59	3.495 (2)	166
C10—H10A···O2^iii^	0.93	2.39	3.302 (2)	167
C11—H11A···O2^iv^	0.93	2.55	3.063 (2)	115
C12—H12C···O2^i^	0.96	2.39	3.334 (2)	168
C17—H17A···O2^iv^	0.93	2.41	3.318 (2)	166
C18—H18A···O3	0.93	2.51	2.892 (2)	105
C12—H12B···Cg1^i^	0.96	2.69	3.515 (2)	144

Symmetry codes: (i) −*x*+2, −*y*+2, −*z*+1;  (ii) *x*, −*y*+3/2, *z*+1/2; (iii) −*x*+2, −*y*+1, −*z*+1; (iv) *x*−1, *y*, *z*.
